# Anthropometric Assessment of Neck Adipose Tissue and Airway Volume Using Multidetector Computed Tomography: An Imaging Approach and Association With Overall Mortality

**DOI:** 10.1097/MD.0000000000001991

**Published:** 2015-11-13

**Authors:** Hillel S. Maresky, Zachary Sharfman, Tomer Ziv-Baran, J.M. Gomori, Laurian Copel, Sigal Tal

**Affiliations:** From the Imaging Department, Assaf Harofeh Medical Center, affiliated with Sackler School of Medicine (HSM, LC, ST); Department of Anatomy, Sackler School of Medicine (HSM, ZS); Department of Epidemiology and Preventative Medicine, School of Public Health, Sackler Faculty of Medicine, Tel Aviv University, Tel Aviv (TZB); and Department of Diagnostic Radiology, Hadassah University Hospital, Hebrew University, Jerusalem, Israel (JMG).

## Abstract

Neck adiposity tissue volume (NATV) accumulation is an indicator for metabolic syndrome and cardiovascular disease (CVD). Neck circumference is a poor measure of NATV, and a quantifier for this entity has not yet been established.

To evaluate volumetric quantification by multidetector computed tomography (MDCT) as a reproducible anthropometric tool to measure NATV and airway volume (AWV).

A total of 519 patients, including a subset of 70 random patients who underwent head and neck CT scanning in our hospital within 1 year (2013), were studied. Included patients were all those undergoing nonenhanced CT (NECT) or CT angiography (CTA). Neck cross-sectional areas (NCSA) were measured at 2 separate levels of the neck, and 3D postprocessing tissue reconstruction was performed, and NATV and AWVs were quantified volumetrically for all patients within the year.

The average NCSA at the level of the soft palate and thyroid cartilage was 22,579 and 14,500 mm^2^, respectively. NATV when compared to the upper and lower levels of NCSA showed correlations of 0.64 and 0.79, respectively (*P* < 0.001). Interobserver analysis showed mean deviations of 0.46% and 0.32% for NATV and AWV, respectively. A strong correlation between NATV and body mass index (BMI) was found (*r* = 0.658, *P* < 0.001), and the top quartile of NATV:AWV patients (out of 519 patients) displayed a statistically significant mortality rate during 670 days of follow-up (d = 7.5%, *P* = 0.032). After adjustment for age and gender, the association between NATV:AWV and mortality was close to significant (*P* = 0.072).

Volumetric quantification of NATV and AWV is a reproducible and prognostic anthropometric tool, as a high NATV:AWV demonstrated a significant risk factor for mortality; future research may further advance our understanding of this phenomenon.

## INTRODUCTION

Obesity represents a major public health concern in the Western world, with more than a third of US and Israeli adults and 17% of young Americans suffering from this disease.^1,2^ Obesity and its comorbid cardiovascular disease (CVD) are thought to make up the bulk of the leading causes of preventable death,^3^ making up 32% of total deaths in Israel.^4^ The body mass index (BMI), as a height-weight calculation, has been losing credibility in the scientific community as an accurate tool to quantify obesity, highlighted by the static BMI trends amongst US adults, but an increasing waist circumference.^5^ The human body is steadily evolving toward a more sedentary and atherogenic form,^6^ but there lacks a proper tool to measure this trend, and we are far from quantifying this pathology.

Neck adipose tissue (NAT) accumulation has been to be shown to be a proxy for obesity. High NAT has been linked more specifically with high visceral adipose tissue (VAT) than subcutaneous adiposity^7^ and is thus thought to be linked most directly with CVD.^8^ Neck circumference measurements have gained ground over BMI in many clinical settings;^9^ however, the tape measure encircles not only fatty tissues and may be misleading, especially in “fit and fat” and “thin-on-the-outside fat-on-the-inside (TOFI)” phenotypes.^10^ Several attempts have been made at specifically quantifying NAT anthropometrically by computed tomography (CT) scanning at single neck levels;^7,11^ however, unlike abdominal adiposity, differences in posture and anatomic variability of the neck impede a single slice from representing one's total neck adipose tissue volume (NATV^12,13^). Volumetric quantification using MRI, while promising, has been hitherto nonreproducible due to the thick slices and slice gaps.^13^ To the best of our knowledge, we are the first to describe a reproducible tool for quantifying total NATV and airway volume (AWV) 3-dimensionally, using multidetector CT (MDCT).

## METHODS

### Study Design: Historical Cohort Study

#### Setting

An 800-bed academic medical center accredited by the Joint Commission International (JCI), serving a mixed urban and rural population of 440,000.

### Study Sample

All patients aged 18 years and above, who underwent CT of the head and neck (NECT and CTA) during the year 2013 at our institution, were included in our study; a subset sample of 70 patients was selected by random number generator for reproducibility studies.

Patients who demonstrated large amounts of oral metal (4 or more dental implants), intubation, pharyngeal hematoma, or head and neck tumors were excluded, due to poor volumetric analysis and unsatisfactory (artifactual) volumetric reconstruction.

### MDCT Scan Protocol

Patients were scanned with Philips MDCT, either Philips iCT 256 or Philips Brilliance 64. The studies that were reconstructed were performed with 2 protocol types: NECT of the neck, and CTA of the head and neck. The NECT protocol was as follows (256 MDCT protocol, and in brackets 64 MDCT): kVP 120, mAs 250 (220) with dose modulation, slice thickness 2 mm, increment 1.5 mm, rotation time 0.5 seconds, field of view (FOV) 250 mm. The CTA protocol was as follows (256 MDCT protocol, and in brackets 64 MDCT): kVP 120, mAs 300 with dose modulation, slice thickness 0.9 mm (1 mm), increment 0.45 mm (0.5 mm), rotation time 0.5 seconds, FOV 220 mm. The protocol included bolus tracking technique (automated tracking of the aortic arch lumen for enhancement during injection of the contrast material bolus). A total of 85 cc of Ultravist 370 mg % (370 mg iodide per 100 cc of solution, Bayer Healthcare) was injected with a rate of 5 cc per sec. This was followed by 20 cc of saline chaser. The scan was initiated automatically 10 seconds after the enhancement of the aortic arch lumen reach 150 HU.

### MDCT Data Analysis

All analyses were performed on a dedicated postprocessing workstation (Philips IntelliSpace Portal v5.0.2.1001). One observer (ZS) performed reconstruction and volumetric analysis on all patients. An experienced and blinded observer (HSM) performed an independent analysis of 70 random volumetric datasets in random order to assess for interobserver variability.

### Quantitative Measure of Neck Cross-Sectional Area (NCSA) NATV and AWV

**FIGURE 1:**
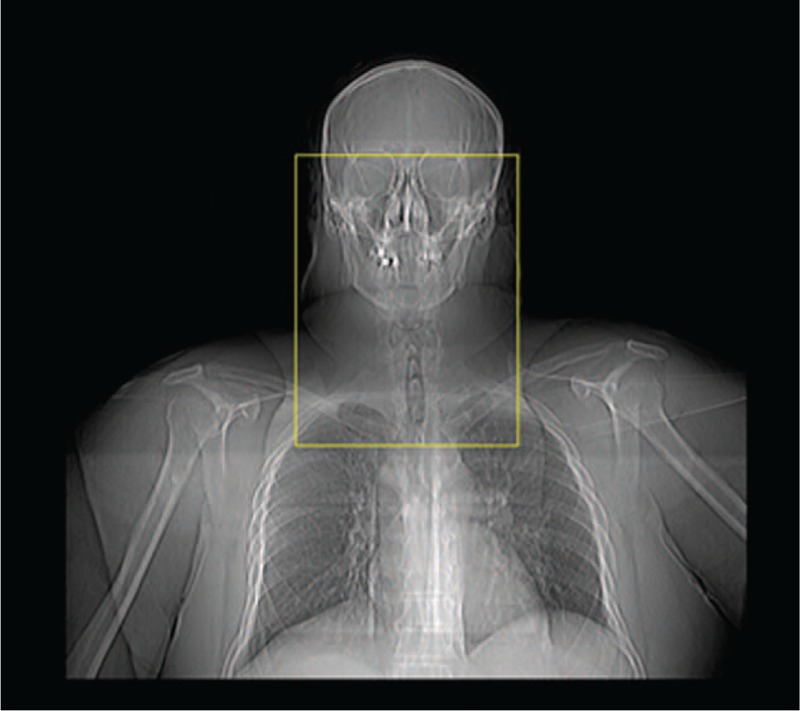
Computed tomography (CT) margins obtained from the scout view: from the roof of the bony orbits cranially, to the sternal angle of lewis inferiorly, and from the mid-clavicular interspace laterally.

**FIGURE 2:**
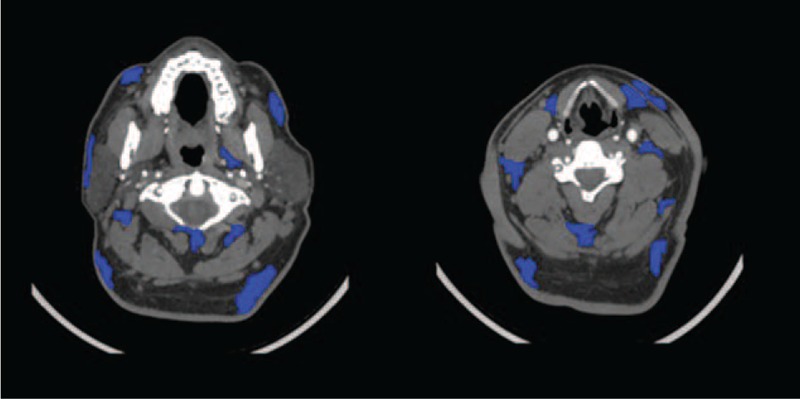
Manual tracing of neck adipose tissue (NAT) compartments at several axial levels.

**FIGURE 3:**
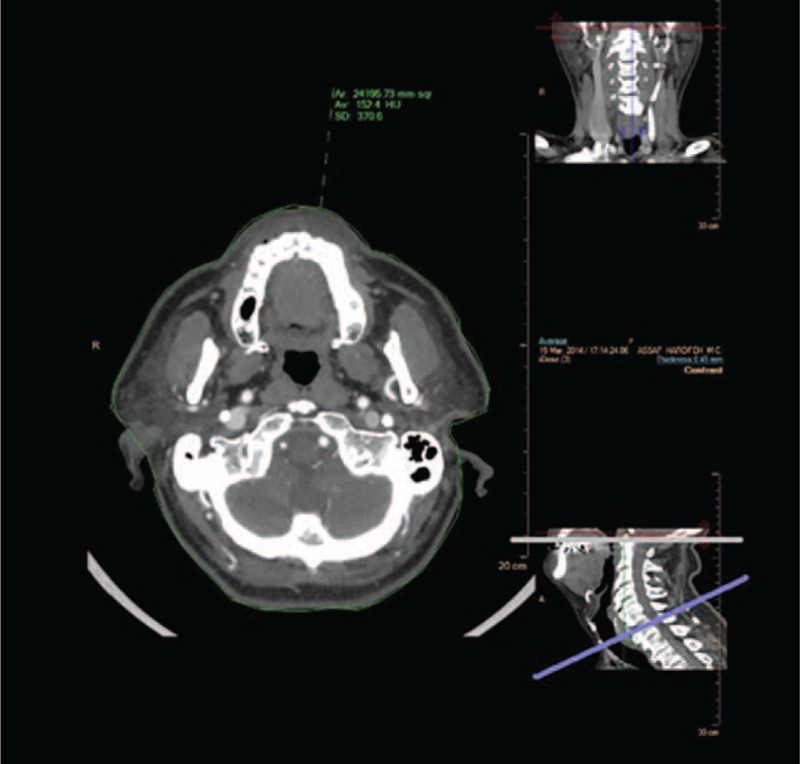
Two levels of neck cross-sectional surface areas (NCSAs): at the level of the soft palate superiorly, and the level of the thyroid cartilage inferiorly. Axial cross-sectional areas were traced semiautomatically, ear cartilage was excluded manually, and expressed in mm^2^.

We measured the NCSA, NATV, and AWV using a dedicated offline workstation (Phillips Intelligence Software, Hewlett-Packard Z600 station with EIZO Display). The NATV was measured across the total imaging volume from the level of roof of orbit cranially to the sternal angle of Lewis caudally, and across the width of intermidclavicular space laterally (Fig. 1). Window width of −150 and −30 HU was defined, with a window center of −90 HU, to identify pixels containing adipose tissue. Twenty adipose tissue ROIs were drawn manually on at least 2 axial slices, and semiautomatically reconstructed and quantified, with manual adjustments if necessary. Adipose tissue was manually traced through all 3 infrahyoid NAT compartments (subcutaneous, posterior, and perivertebral compartments^7^), using tissue segmentation on coronal and sagital sections until agreement with the semiautomatic segmentation (Fig. 2). AWV was similarly manually traced and automatically reconstructed from the retro-pharynx at the level of the hard palate cranially (the oropharynx was excluded to avoid sampling error due to metallic artifacts from dental implants), until and including the first tracheal ring caudally. The NCSA (mm^2^) was measured using a single slice (5 mm thickness), perpendicular to the spine, at the level of the caudal tip of the soft palate cranially, and at the level of the cranial tip of the hyoid cartilage caudally (Fig. 3). The average time for image analysis was 17 minutes per patient.

### Patient Characteristics

Data on age, sex, and BMI were extracted from the 70 patient subset's medical records. BMI calculations were divided into 3 categories, based on WHO criteria:^14^ normal and under-weight, overweight, and obese (Class I Obesity and Class II Obesity), using cutoff values of 25 and 30. All patients were followed up to 670 days.

### Sample Size

In order to evaluate an intraclass correlation coefficient (ICC) of 0.9 with a 95% confidence interval (CI) width of 0.1 (with 2 observations per patient), 61 patients were required (WinPepi Version 11.43).^15^

### Statistical Analysis

Categorical variables were described using frequency and percentage. Continuous variables were evaluated for normal distribution using Histogram and Q–Q plots. Normally distributed continuous variables were described using mean (standard deviation, SD), and nonnormally distributed continuous variables were also described by median (interquartile range, IQR). Interobserver agreement was described using Bland–Altman plots; a fixed bias was evaluated using one sample *t*-test. Reliability between observers was evaluated using ICC for absolute agreement. Spearman correlation coefficient was used to assess the relationship between NATV and AWV with BMI and NCSAs. The Kruskal–Wallis test and Mann–Whitney *U* test were used to evaluate the difference in NATV and AWV between BMI categories. Kaplan–Meier curves and Log-Rank tests were used to evaluate mortality during follow-up in patients in the upper quartile of NAT:AWV versus lower quartiles. Univariate Cox regression was used to evaluate the crude and age and gender adjusted association between NAT:AWV and all-cause mortality. Hazard ratio with 95% CI was reported. All statistical analysis was performed using SPSS v22; *P* < 0.05 was considered statistically significant.

### Ethics

This study was approved by the hospital's institutional review boards (file number 15/14), waiving written informed consent from all patients in this study.

## RESULTS

A total of 519 patients were included in the study, with a random subset of 70.

### Interobeserver Agreement

**FIGURE 4:**
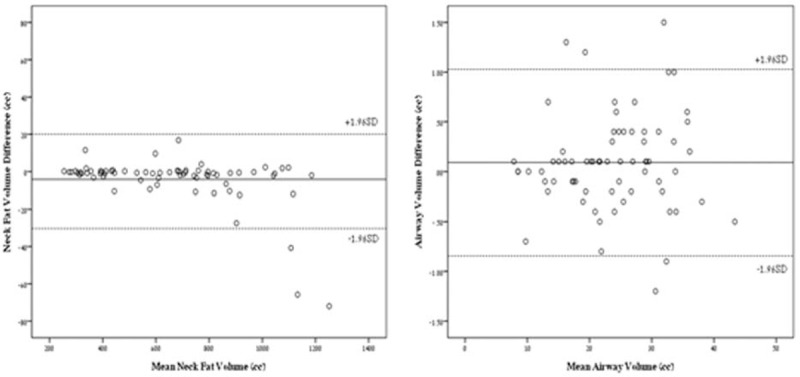
Bland–Altman plots for interobserver NATV and AWV. AWV = airway volume, NATV = neck adipose tissue volume.

Of the subset, 25 patients underwent NECT (35.7%), and 45 underwent CTA (64.3%). Indications for NECT included aspiration of foreign body (22.9%), trauma (3.5%), and cervicalgia (2.9%), while indications for CTA were stroke-like symptoms (58.6%) and suspected carotid artery dissection (5.7%). Twenty five patients (35.7%) were female and 45 were male (64.3%). The median age was 56.8 years (range 14–92.8). A third of the patients (35.4%) were of normal or underweight, 29.2% were overweight, and 35.4% were obese. The mean NATV was 660 cc (SD = 266), and median NATV was 681 cc (IQR = 406–838). The mean NATV interobserver difference was 4.17 cc (SD = 13.35), and the median NATV interobserver difference was −0.75 cc (IQR = −2.8–0.2), *P* = 0.011 (Fig. 4). The difference represents a mean discrepancy of 0.42% between observers. There was a near absolute agreement between observers of NATV (ICC = 0.999, *P* < 0.001).

The mean AWV was 23.6 cc (SD = 8.1), and median AWV was 23.9 cc (IQR = 17.4–29.8). The mean AWV interobserver difference was 0.09 cc (SD = 0.47), and the median AWV interobserver difference was 0.1 (IQR = −0.2–0.3), *P* = 0.114 (Fig. 4). The difference represents a mean discrepancy of 0.36% between observers. There was a near absolute agreement between observers of AWV (ICC = 0.998, *P* < 0.001). Average reconstruction time per patient for both NATV and AWV was 17 minutes.

**FIGURE 5:**
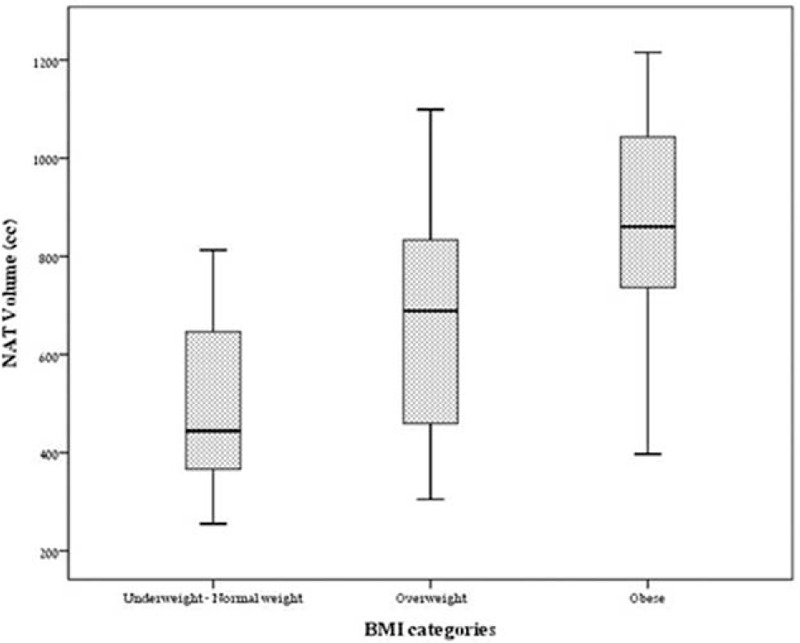
NATV distribution according to WHO BMI categories. BMI = body mass index, NATV = neck adipose tissue volume, WHO = World Health Organisation.

A strong correlation between NATV and BMI was observed (*r* = 0.658, *P* < 0.001). All 3 WHO BMI categories were well represented based upon NATV calculation (Fig. 5). The median NATV in the BMI category of normal and under-weight was 444 cc (IQR 341–685), while in the overweight category the median was 689 cc (IQR 412–877), and in the obese category 864 (IQR 713–1047), *P* < 0.001. No statistically significant association between AWV and BMI was found (*r* = −0.117, *P* = 0.353).

A very strong association between NATV and NCSAs was observed at the level of the thyroid cartilage (*r* = 0.791, *P* < 0.001), and as well at the level of the soft palate (Fig. 3, *r* = 0.645, *P* < 0.001). AW was inversely proportionate to both NATV and BMI, however not statistically significant (*r* = −0.34, −0.28; *P* = 0.18, 0.11, respectively).

### Mortality

**FIGURE 7:**
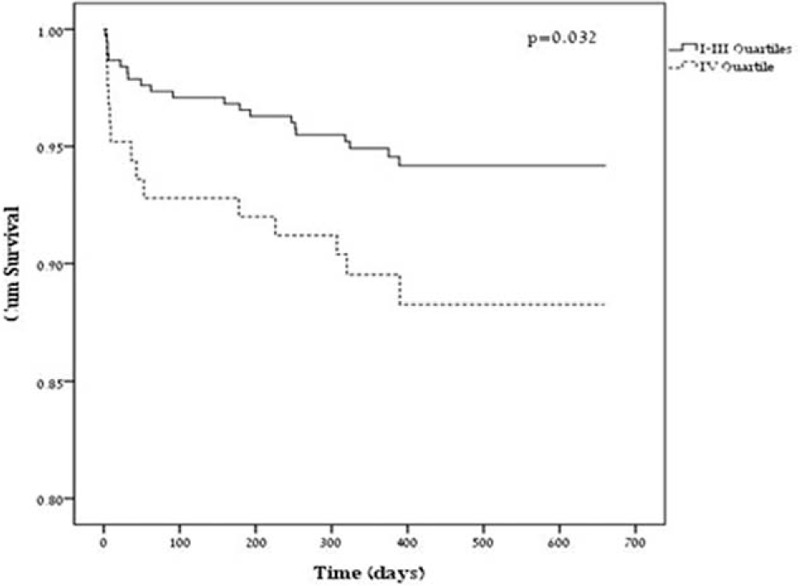
Cumulative 670 days survival follow-up through NATV:AWV quartiles. AWV = airway volume, NATV = neck adipose tissue volume.

A total of 419 patients (82.3%) underwent CTA. Two hundred seventy nine patients (54.8%) were male. The median age was 61.3 years (IQR 49.5–71.3). Thirty five patients in all (6.9%) died during the follow-up. The median follow-up was 443 days (IQR 352–541). When calculated as a fraction and expressed as a ratio; the median NAT:AWV was 29.6 (IQR 20.2–42.8). Higher NAT:AWV was associated with mortality (HR = 1.012, 95% CI 1.001–1.023, *P* = 0.032). After adjustment for age and gender, the association between NATV:AWV and mortality was close to significant (HR = 1.01, 95% CI 0.999–1.024, *P* = 0.072) Fourteen patients (11.2%) from the upper NAT:AWV quartile died during the follow-up, while only 21 patients (5.6%) from the lower 3 quartiles died (HR = 2.09, 95% CI 1.07–4.12, *P* = 0.032, Fig. 7).

Higher BMI was not associated with mortality (HR = 1.05, 95% CI 0.98–1.13, *P* = 0.180). This association was even less significant when adjusted for age and gender (HR = 1.04, 95% CI 0.97–1.12, *P* = 0.295). Twelve patients (6.6%) from the under and normal weight categories died during the follow-up, while 13 patients (6.4%) from the overweight categories, and 9 (7.9%) from the obese categories died, with no significant difference (*P* = 0.862). This lack of significance was not changed even after adjustment for age and gender (*P* = 0.896).

## DISCUSSION

Our anthropometric pilot study of NATV and AWV demonstrates excellent interobserver reproducibility of MDCT-based volumetric quantification. We also demonstrate a clear correlation between NATV and NCSA at 2 separate levels, with a stronger correlation observed at the NCSA level of thyroid cartilage. This finding supports the use of tape measure circumference calculations at the level of the thyroid cartilage as a gross NATV evaluator, in a supine patient in the primary care setting.^16^

**FIGURE 6:**
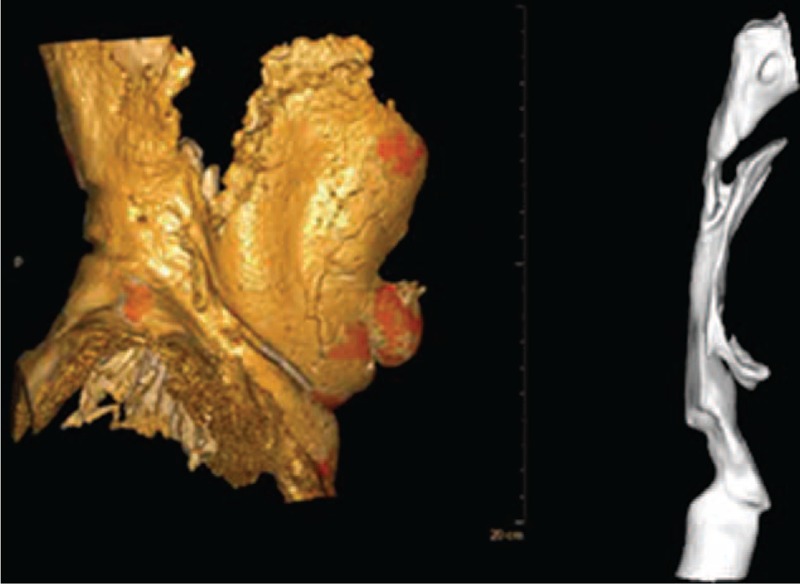
Illustration of NATV (A) and AWV (B) reconstructed 3-dimensionally. AWV = airway volume, NATV = neck adipose tissue volume.

Neck circumference and NAT have been shown to represent a high risk factor for CVD and overall mortality using 2-dimensional techniques.^7,14^ Single-slice imaging, however, is limited to the slice selected, and tape measure calculations are limited to the position of the patient's neck:^17^ if the patient's neck is lordotic, more NATV is shifted to the posterior compartments, and vice versa. Unlike abdominal adiposity, neck flexion and extension greatly impede the reproducibility of 2-dimensional NAT or area quantification.^13^ In our report, we describe a novel and reproducible 3-dimensional technique which overcomes the limitations of 2-dimensional analysis by using large “slack” margins, thereby deriving accurate quantitative and volumetric measurement of overall NATV (Fig. 6).

We suggest the use of total NATV quantification as a reliable tool to measure NAT. Only total NATV quantification with extra-cervical “slack” above and below the anatomic triangles of the neck takes into account this limitation.^18^ Higher indexes of error were observed at higher cervical adiposity levels (in cases of NATV above 1050 cc), possibly due to the substantial shifting of NAT into the predefined “slack” areas (bony roof of the orbits superiorly, and sternal angle of Lewis inferiorly), and thus may limit the ability to quantify mega-NATV using our study's technique. AWV (Fig. 6) has only recently been studied volumetrically, by Chooi and Marcus,^19^ and we highly endorse their work. Furthermore, our data suggests that height-weight anthropometric measures such as BMI are less specific tools than neck measurements such as NATV or AWV to predict mortality.

Limitations include the small subset of 70 patients of whom full clinical data was collected and analyzed. NATV trends followed the expected age and sex-related differences as expected with BMI,^14^ as NATV was closely correlated with BMI (*r* = 0.66, *P* < 0.001). An inverse relationship between NATV and AWV was also observed (*r* = −0.34), likely due the anatomic NATV compressing the hard airway;^20^ however, due to the small subset of 70 patients the null hypothesis may not be rejected (*P* = 0.11). This subset of 70 patients was selected randomly from all patients undergoing head and neck CT at our institution within the year 2013 for the purpose of establishing interobserver variability, and in order to avoid selection bias. We do not have information regarding the cause of mortality of all patients, and instead demonstrate a correlation of high NAT:AWV with all-cause mortality. Thus, we cannot be sure that the mortality was directly correlated to obesity-related diseases. Due to the retrospective nature of our study, we do not have data on comorbidities; however, this is a pilot study to suggest an imaging tool for future prospective studies that may control for possible comorbidities.

NAT accumulation has been shown to be a good proxy for VAT,^10,17^ and NATV measurement could represent a “tip of the iceberg” approach to quantifying a patient's visceral adiposity and therefore CVD risk.^7^ The speed, simplicity, and reliability of the examination could offer an alternative to total body MRI scanning for adiposity, which is an expensive and timely procedure.^13^ Some research has shown the use of AWV quantification, and its correlation to sleep apnea;^20^ however, the relationship between NATV and AWV has not yet been fully studied volumetrically. The anatomic relation between neck adiposity and AWV was indicative in our study for overall morbidity in a large sample of patients (n = 519).

Anthropometric studies of neck girth and adiposity have alluded to the promising role of NATV quantification as an indication of obesity and sleep apnea,^20,21^ as well as further understanding the potential relationship between imaging studies as anthropometric tools and metabolically important fat.^22^ Future clinical studies are warranted to further understand total NATV and AWV not only as a measure of cervical adiposity and as a risk factor for overall mortality, but also to investigate the wide array of potentially associated comorbidities such as CVD, sleep apnea, and stroke.
